# Transcriptional Control of Metastasis by Integrated Stress Response Signaling

**DOI:** 10.3389/fonc.2021.770843

**Published:** 2021-10-20

**Authors:** Si Lu, Li-Xian Yang, Zi-Jian Cao, Jiang-Sha Zhao, Jia You, Yu-Xiong Feng

**Affiliations:** ^1^ Zhejiang Provincial Key Laboratory of Pancreatic Disease, First Affiliated Hospital, Institute of Translational Medicine, Zhejiang University School of Medicine, Hangzhou, China; ^2^ Cancer Center, Zhejiang University, Hangzhou, China; ^3^ School of Life Sciences, Westlake University, Hangzhou, China

**Keywords:** metastasis, transcription, integrated stress response (ISR), cancer, EMT

## Abstract

As a central cellular program to sense and transduce stress signals, the integrated stress response (ISR) pathway has been implicated in cancer initiation and progression. Depending on the genetic mutation landscape, cellular context, and differentiation states, there are emerging pieces of evidence showing that blockage of the ISR can selectively and effectively shift the balance of cancer cells toward apoptosis, rendering the ISR a promising target in cancer therapy. Going beyond its pro-survival functions, the ISR can also influence metastasis, especially *via* proteostasis-independent mechanisms. In particular, ISR can modulate metastasis *via* transcriptional reprogramming, in the help of essential transcription factors. In this review, we summarized the current understandings of ISR in cancer metastasis from the perspective of transcriptional regulation.

## Introduction

Development of secondary lesions in organs or tissues that are physically disconnected with the original primary cancer is termed metastasis. As a systemic disease, metastasis requires cancer cells to leave their primary organ, circulate in the bloodstream, endure the pressure of the vasculature, adapt to the new microenvironment in a secondary site, survive the hostile battle against immune cells, and finally repopulate in the new organ (1). Once developed and flourished, metastasis is usually irresistible and life-threatening (2). Despite the advances in the basic and clinical research of cancer, the vast majority of patients suffering metastatic diseases invariably confront a terminal illness that is incurable by current therapeutic approaches. Metastasis is the cause of 90% of deaths from solid tumors (3). In contrast to the large body of findings that have revealed the detailed pathogenetic mechanisms leading to formation of primary tumors, the biological mysteries of metastatic disease remain poorly understood. Unlike the well-documented principles that can well define the uncontrolled growth of tumors, relatively few mechanisms have emerged that would fully explain how diverse types of metastases arise and how similar or different each may be relative to the behavior of its corresponding primary tumor. Key oncogenes and tumor suppressors have been discovered and appreciated in the development of primary tumors (4, 5), yet few, if any, metastasis-specific gene mutations or genomic disorders have been identified and functionally validated (6–8).

The integrated stress response (ISR) is a conserved network of signaling pathways that helps the organism to adapt to an ever-changing environment and maintain the integrity and health of the host (9, 10). In response to a variety of environmental and pathological conditions, including nutrient deprivation, oxidative stress, heat shock, protein homeostasis defects, and viral infection, the ISR copes these hostile stimuli and restores the cellular balance by reprogramming gene expression and protein translation (11–13). It has also been demonstrated that the ISR can be triggered by oncogene activation during tumorigenesis in human cancers. Invariably, these various stresses are sensed by four cellular kinases, including PERK, GCN2, PKR, and HRI, that can converge to phosphorylate a single serine on eIF2 (eukaryotic translation initiation factor 2) (14, 15). Upon phosphorylation, p-eIF2 can reduce protein synthesis by blocking the function of eIF2’s guanine nucleotide exchange factor termed eIF2B. Interestingly however, while shutting down the production of most proteins, p-eIF2 promotes the translation of certain specific mRNAs, including key transcription factors, such as ATF4. It has been well-documented that these mRNAs contain inhibitory ORFs in their 5’-UTRs that can inhibit translation initiation at their canonical translation start sites (16). Through a simultaneous down-regulation of general translation and up-regulation of the synthesis of particular proteins that can reset gene transcription, the ISR aims to rebuild homeostasis in order to maintain the integrity of the host. However, if the stresses are too severe to be managed or resolved, the ISR can eliminate the damaged cells by executing programmed cell death.

## Biological Cascades of Metastasis

The formation of clinically detectable metastasis is the ultimate, end result of a series of stochastic events that first allow cancer cells to disperse and survive in distant sites and later to grow as secondary tumors. The sequence of metastasis steps starts with the departure of cancer cells from the primary tumor and ends in the formation of clinically detectable macro-metastases in the target organs. Apparently, this is a multi-step, long-term adventure for cancer cells. In general, it comprises steps of local invasion (or so called invasive transition), entry into the circulation, travel along the vasculature, arrest at secondary sites, extravasation, and colonization (metastatic outgrowth) (17).

Invasion represents the very first step and a pre-requisite to metastatic dissemination. Cancer cells are required to take a plastic phenotype to complete an invasive transition. A diverse set of factors and molecular circuits can equip the cancer cells of plasticity and trigger this transition. Originally discovered as an essential transdifferentiation program in development and tissue repair, epithelial-mesenchymal transition (EMT) is known as the key mechanism in promotion of cancer invasion. While cancer cells at the boarder of cancer-normal tissue can invade in a single-cell migration or collective migration manner, they usually display a mesenchymally transdifferentiated cell state, or at least a partial (hybrid) EMT (18). In addition to EMT, an inflammatory microenvironment, or a systemic inflammatory response, can also trigger the invasive transition of otherwise stationary cancer cells in the primary organ (19). Related to this, certain types of normal cells within the microenvironment, for example, cancer-associated fibroblast (CAF), can be co-opted, or educated, by the cancer cells to promote cancer cell invasion (20, 21).

Dissemination comes after invasion. After entering into the bloodstream, tumor cells must circulate and survive the hostile environment before they can extravasate and form secondary tumors. Circulating tumor cells (CTCs) represent an intermediate stage of metastasis (22). While some CTCs passively enter the bloodstream, CTCs derived from actively invading cells acquire key properties required for metastatic spread while still facing significant subsequent barriers to generate a metastatic lesion. CTCs can circulate as single cells or cell clusters, with the latter appearing to have increased metastatic potential (23). Most CTCs die in the circulation, likely from a combination of physical stress, oxidative stress, anoikis, and the lack of growth factors and cytokines. To survive, CTCs must actively extravasate into the surrounding tissue or, in a more passive manner, become clogged or lodged in a capillary bed.

Once the cancer cells exit the bloodstream, they may begin to divide and attempt to complete the last step of metastasis, colonization. Colonization can be divided into steps: formation of micro-metastasis, latency in the distant organ, re-activation of growth in the latent micro-metastases, and a final, life-threatening, aggressive overtaking of the target tissue (24). In most cases, the disseminated tumor cells may reach an equilibrium between active proliferation and cell death that prevents their outgrowth, and finally eliminated by the host immune system. Only very few of them can enter the dormancy but appear to retain the ability to ultimately grow into a detectable metastatic lesion. To progress to this final step, it requires several specialized functions including cancer cell-autonomous functions and the cooption of various components of the target tissue stroma (24).

## Stresses in the Metastatic Cascades

Metastasis is a highly inefficient process. Over a million cancer cells can be released by the primary lesion into the circulation every day, but only a very minor portion of them is capable of colonizing a distant organ, even fewer can flourish into clinical detectable macro-metastasis (25). For long, this phenomenon is explained by the concept that metastasis emerges from the somatic evolution of a genetically diversified cancer cell population under selective pressures (1). In another word, it suggests that metastasis is similar, at least phenotypically, to an evolutionary process that enlists selection of genetically heterogeneous lineages of cancer cells. However, the recent efforts of genomic sequencing of metastatic lesions, as well as their counterparts at the primary sites, did not reveal metastasis-specific genes that can drive or sustain metastatic outgrowth (6). While the evidence of selection during metastasis is obvious, the cellular mechanism underlying the selection is unclear.

The metastatic cascade progresses in biological environment that is drastically different than where it initiates at the first place. Therefore, metastatic tumor cells will inevitably encounter numerous stresses that force them to develop circuits or pathways to obtain improved aptness along the metastatic marathon. It has been proposed that instead of behaving as traditional driver oncogenes, the so-called “metastasis adaptiveness genes” can increase the metastatic potential and successful rates by relieving stresses that are not encountered in physiological conditions (26). Thus, only a small subset of cancer cells that are capable of adapting to the hostile, stressful metastatic cascade can complete the whole process and develop into macro-metastasis. Going beyond their functions in anti-stress, some stress response pathways can directly drive metastasis by increasing cell mobility, invasion, and additional functions related to metastatic outgrowth. It is increasingly appreciated that metastasis can be steered by the mechanisms that tumor develop to cope with the stresses that they encounter in the metastatic cascade. In this review, we will primarily focus on one the major stress response pathway, the integrated stress response (ISR) pathway.

## Mechanisms of ISR Activation

**Figure 1:**
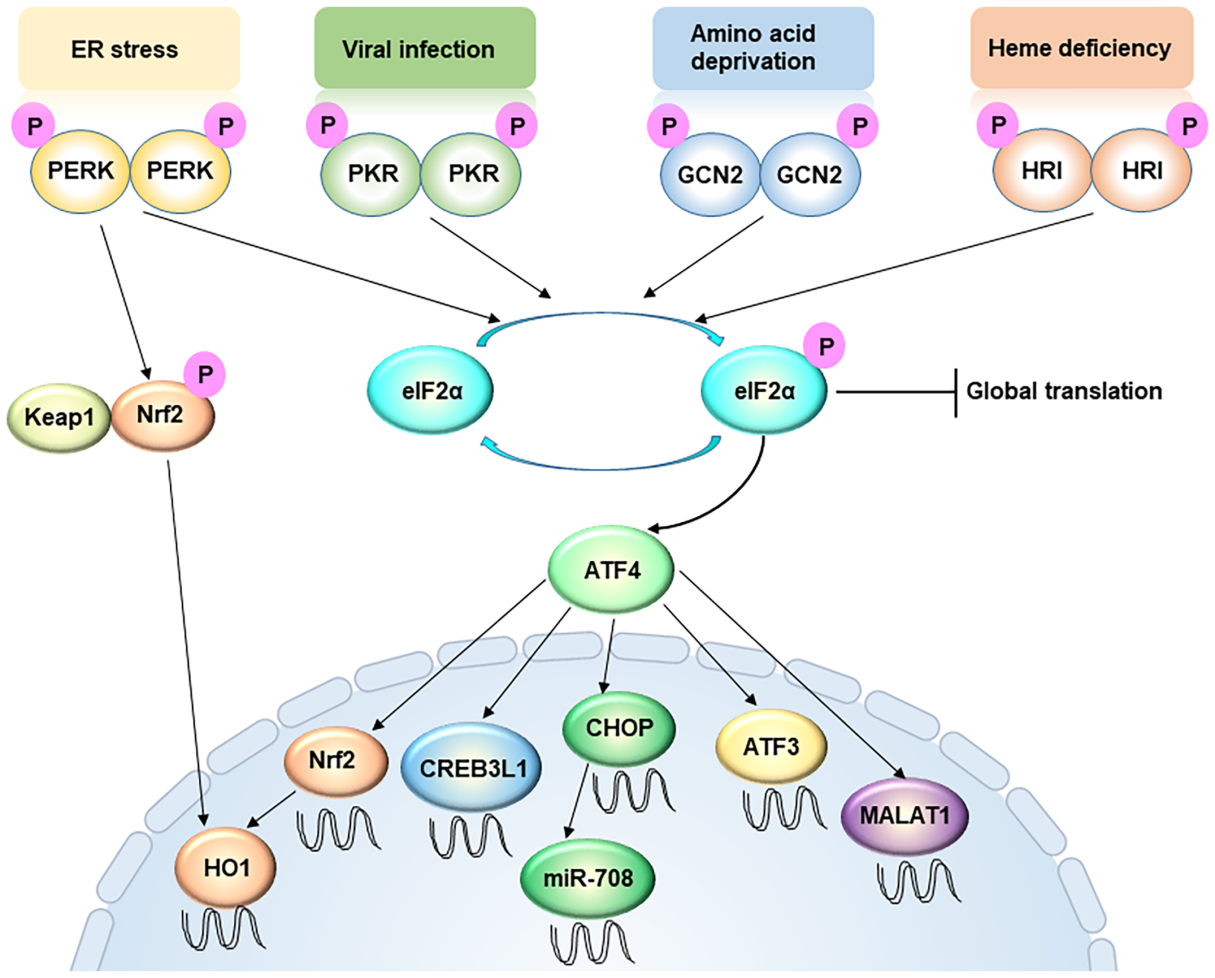
Integrated stress response signaling, from a metastasis perspective. ER stress, viral infection, amino acid deprivation, and heme deficiency activate PERK, PKR, GCN2 and HRI, respectively. These four kinases converge on phosphorylation of eIF2α, an initiation factor of protein synthesis, which leads to global translation attenuation but promotes the translation of certain specific mRNAs, including ATF4. The PERK-ATF4 pathway is involved in oxidative resistance in two manners. On one hand, PERK directly phosphorylates Nrf2, causing the latter to disassociate with Keap1 and translocate into the nucleus. On the other hand, ATF4 directly drive Nrf2 transcription. Nuclear accumulation of Nrf2 triggers the transcription of redox homeostasis genes, including HO-1. ATF4 also enhances CREB3L1 transcription to promote cancer cell migration and invasion. However, ATF3, another target of ATF4, usually represses cell migration and invasion. In addition, LncRNA MALAT1 is a target of ATF4, which is critical for extravasation and subsequent outgrowth of cancer cells. MiR-708 can be up-regulated by CHOP, a downstream target of ATF4.

The ISR responds to a variety of different stress conditions that interfere with the cellular homeostasis (**Figure 1**) (27). So far, these stresses are sensed, processed, and transduced by four different kinases that phosphorylate Ser51 of eIF2α to activate the ISR. In both yeast and mammalian cells, the ancestral kinase GCN2 responds to amino acid deprivation, whereas in metazoans the repertoire of kinases expands to include PERK, HRI, and PKR. All four kinases contain both conserved kinase domains and divergent regulatory domains that enable them to respond to different stimuli (27). Stress signals sensed by the regulatory domains cause kinase activation by dimerization and trans-autophosphorylation (28, 29). When extracellular amino acids are deprived or low, intracellular deacylated His-tRNA will increase and subsequently bind and activate GCN2. GCN2 can also be activated by other stresses, including serum starvation and oxidative stress (30). PERK is a transmembrane protein locating on the ER membrane, and mediates UPR (28). In specific, the N-terminal domain of the PERK protein resides in the ER lumen and associates with the chaperone protein BiP. During ER stress, the accumulated misfolded/unfolded protein can replace BiP and directly activate PERK by binding to its ER luminal domain. PKR contains a double-stranded RNA (dsRNA) binding domain, therefore viral dsRNA and secondary structures resembling dsRNA on mRNAs can directly activate PKR (31). HRI contains an N-terminal heme-binding domain so that a low level of heme can directly trigger the activation of HRI. For long HRI was considered a specialized protein in erythroid cells and mainly involving in hemoglobin synthesis (32). Recent studies suggest that HRI is actually widely expressed in several cell types other than red blood cells, and responds to multiple other forms of stresses, such as oxidative and mitochondrial stress (33, 34). Therefore, the mechanism of ISR activation appears increasing complex and intertwined.

## ISR, Protein Translation, and Cancer

The link between ISR and cancer has been long appreciated. Not surprisingly, as a pathological process, which inevitably interferes with normal homeostasis, cancer development enlists a great number of different types of stresses. Oncogene activation, including cancer-specific mutation or overexpression, could elicit stresses on transformed cells that normal cells usually do not encounter. For instance, mutations in tumor suppressor genes, such as PTEN, or the over-production of oncogenes, such as c-MYC, results in increased protein synthesis, which can saturate the proteostasis machinery and activate the ISR (35, 36). For virus-related tumors, such as liver and cervical cancers, chronic viral infection can be sensed by PKR and activates ISR (37). During tumor expansion, nutrient deficiency is a major hurdle that constrains the uncontrolled growth of tumors. ISR is activated upon nutrient deprivation and could be pro-survival and pro-apoptotic for cancer cells (27). In addition, the balance between oxidation and reduction of cancer cells usually goes awry, leading to cellular stresses including oxidative stress, unfolded protein stress, and mitochondrial stress (38). These stresses will converge to activate ISR and, in turn, greatly influence tumor development.

Aberrant cell survival and reduced cell death are hallmarks of cancer cell. For long, the role of ISR in cancer development has been largely linked to and explained by the function of protein translation regulation on cell death/survival balance. Indeed, the ISR kinases have been implicated in cancer regarding cell death and proliferation. Activation of the ISR by PERK promotes tumor initiation and progression (35). Consistently, in PTEN loss and MYC-driven prostate cancer models, the PERK branch of the ISR is activated and limits protein synthesis rates. Paradoxically, inhibition of either the PKR branch of the ISR or phosphorylation of eIF2 led to transformation of mouse fibroblasts and increased tumor formation in immune-deficient mice (39). These seemingly contradictory results could be explained by the complexity of ISR functions, which manage both pro-survival and pro-death mechanisms, including the activation of NFκB, PI3K, and JNK pathways (40). In addition, the dependency of cancer cells on protein translation, as well as the tolerance of cancer cells to protein synthesis restriction, varies greatly among different types of cancers.

## Transcriptional Control of Metastasis by ISR

While the majority of current studies attribute the role of ISR in cancer to its function in protein translation regulation, emerging evidences demonstrate that ISR could affect tumor progression *via* translation-independent mechanisms. Metastasis, as a complicated, multi-step process, requires the many faces of the ISR to support and facilitate cancer cells to survive this challenging journey. In particular, the role of the ISR in transcriptional reprogramming has been increasingly appreciated (**Figure 1**).

## ISR Transcriptionally Controls Cancer Cell Motility and Invasiveness

Increased motility, along with heightened invasiveness, represents the first hallmark that allows the initiation of metastasis. Many of the ISR-related transcription factors regulate cell migration and invasion. Selectively activated and upregulated by the ISR, ATF4 is an essential transcription factor that transduces the signal of ISR. In addition to its canonical roles, ATF4 can promote migration and invasion in different types of cancers, including breast cancer, esophageal cancers, and bladder cancers (41, 42). This critical roles of ATF4 in cancer can explain the pro-metastatic function of PERK in breast cancers, as PERK depletion effectively mitigates the metastatic lesions developed in a Her2-driven breast cancer model (43). A recent study suggested that ATF4 is downstream of the TGFβ signaling pathway and facilitates the latter’s pro-migratory function in triple negative breast cancers (TNBC) (44). Modulation of extracellular matrix (ECM) has been attributed to the mechanism of ATF4-driven invasion, since ATF4, with the help of Fra-1, directly drives the expression of CREB3L1, a transcription factor critical in remodeling ECM (45). CREB3L1 can regulate the FAK signaling during cancer cell migration and invasion. This CREB3L1-mediated signaling program is specifically activated in breast cancer cells when compared to their normal counterparts, representing a non-stress-dependent mechanism of the PERK branch of the ISR in cancer metastasis (45, 46). Importantly, the unique mode of activation, namely proteolytic cleavage, of CREB3L1 announces itself as potentially targetable transcription factor in treating metastasis (45).

While CREB3L1 can mediate the function of ATF4 in promoting metastasis, other downstream factors of ATF4 seem to antagonize the pro-invasive role of ATF4. ATF3 is a classical ISR TF that is regulated by ATF4 during ISR activation (47). However, ATF3 has been reported to inhibit migration and invasion in multiple types of cancers, including squamous cell carcinoma, glioma, colon cancer and renal cell carcinoma (48–50). Interestingly, the anti-migration function of ATF3 is not linked to its canonical role in stress signaling or proteostasis.

## ISR Facilitates Distant Colonization

While the role of many ISR-TFs in the initiation step of metastasis has been well studied, their functions in the steps onwards are much less appreciated. Surviving a non-adherent condition in blood stream is a prerequisite of cancer cells to proceed their journey of metastasis. In fibrosarcoma, ATF4 is induced upon detachment and suspension of cancer cell, and it is critical to protect cancer cells from anoikis. Mechanistically, ATF4 directly drives NRF2 transcription and nuclear accumulation, which in turn triggers the transcription of redox homeostasis genes, including HO-1 (51). In fact, NRF2 is critical in regulating the expression of many important genes involved in oxidative stress response (52). This important function of ATF4 makes it indispensable for successful colonization of fibrosarcoma cells in the lung of the mouse models. It is known that an appropriate anti-oxidant response is essential for human melanoma cells to survive distant metastasis, the potential role of other ISR-TFs should be further investigated from this perspective.

In addition to regulating gene transcription *via* its canonical downstream factors, the ISR can modulate metastatic colonization and outgrowth *via* certain LncRNAs. Malat1 is a LncRNA induced upon PERK activation (53). In a human lung cancer model, Malat1 is critical for extravasation and subsequent outgrowth of cancer cells. Importantly, inhibition of this LncRNA by its antisense oligonucleotides (ASO) can effectively abolish lung metastasis (54). In addition, miR-708, a CHOP-regulated miRNA (55), also serves as a potential therapeutic agent for breast cancer metastasis as it can regulate calcium-mediated cell migration (56). It will be of interest to further investigate the potential crosstalk between the ISR-TFs and ISR-LncRNAs in metastasis regulation.

## ISR and Metastasis-Related Cell State Transition

Other than obtaining different capacities for metastasis by activating different signal pathways, cancer cells can equip themselves with metastatic potential by turning on cell-state transition programs, which can comprehensively and robustly change the cell phenotype in a coordinated, efficient manner. EMT represents one of these programs. It has been shown that the PERK-eIF2α-ATF4 cascade is activated and implicated during an EMT (57). Tumor cells acquiring an EMT, characterized as loss of epithelial markers and gain of mesenchymal markers, become more invasive, metastatic, stem-like and drug-resistant. The PERK branch of the UPR is activated upon EMT-induction, likely due to the reason that cancer cells of an EMT state hyper-activate the secretory pathway, which requires the UPR to accommodate an increase in both protein production and secretion. The correlation between EMT and PERK-ATF4 activation is also confirmed in primary breast cancer, colon cancer, lung cancer, as well as metastatic cancers spanning hundreds of clinical samples. In addition to the canonical PERK-ATF4 pathway, the non-canonical PERK-NRF2 pathway is also activated in cancer cells that undergo dedifferentiation. The change of NRF2 signal can render cancer cells a higher ability to resist chemotherapy drugs, which is a hallmark of many metastatic diseases (58, 59).

## Conclusion and Perspective

It remains a central question in cancer research how cancer cells acquire the competence to colonize distant organs. Tumors can release large numbers of cancer cells into the circulation, but only a small proportion of these cells survive on infiltrating distant organs and even fewer form clinically meaningful metastases. Many predictive gene signatures and specific mediators of metastasis have been identified, yet how cancer cells acquire these traits has remained obscure. Given the highly hostile nature of the metastatic cascades, the ISR signaling is activated and participates in every single steps during metastatic progression. The discoveries of essential transcription factors induced by the ISR open a window on an entirely new avenue of investigation of metastasis, and will provide valuable potential targets for treating this currently incurable disease.

## Author Contributions

Y-XF conceived the concept of this manuscript. SL, L-XY, and Z-JC collected materials and analyzed literatures related to this review topic. J-SZ, JY, and Y-XF wrote the manuscript. All authors contributed to the article and approved the submitted version.

## Funding

This study was funded by the National Natural Science Foundation of China (31871369, Y-XF) and the Zhejiang Provincial Natural Science Foundation of China (LD19H160002, Y-XF).

## Conflict of Interest

The authors declare that the research was conducted in the absence of any commercial or financial relationships that could be construed as a potential conflict of interest.

## Publisher’s Note

All claims expressed in this article are solely those of the authors and do not necessarily represent those of their affiliated organizations, or those of the publisher, the editors and the reviewers. Any product that may be evaluated in this article, or claim that may be made by its manufacturer, is not guaranteed or endorsed by the publisher.
